# Probiotics and Alcoholic Liver Disease: Treatment and Potential Mechanisms

**DOI:** 10.1155/2016/5491465

**Published:** 2015-12-29

**Authors:** Fengyuan Li, Kangmin Duan, Cuiling Wang, Craig McClain, Wenke Feng

**Affiliations:** ^1^College of Life Sciences, Northwest University, Xi'an, Shaanxi 710069, China; ^2^School of Pharmaceutical Sciences, Wenzhou Medical University, Wenzhou, Zhejiang 325035, China; ^3^Departments of Medicine, Pharmacology and Toxicology, University of Louisville School of Medicine, Louisville, KY 40202, USA; ^4^Robley Rex Veterans Affairs Medical Center, Louisville, KY 40202, USA

## Abstract

Despite extensive research, alcohol remains one of the most common causes of liver disease in the United States. Alcoholic liver disease (ALD) encompasses a broad spectrum of disorders, including steatosis, steatohepatitis, and cirrhosis. Although many agents and approaches have been tested in patients with ALD and in animals with experimental ALD in the past, there is still no FDA (Food and Drug Administration) approved therapy for any stage of ALD. With the increasing recognition of the importance of gut microbiota in the onset and development of a variety of diseases, the potential use of probiotics in ALD is receiving increasing investigative and clinical attention. In this review, we summarize recent studies on probiotic intervention in the prevention and treatment of ALD in experimental animal models and patients. Potential mechanisms underlying the probiotic function are also discussed.

## 1. Introduction

Chronic alcohol consumption is a major cause of liver injury. Alcoholic liver disease (ALD) encompasses a broad spectrum of stages including fatty liver, inflammation, fibrosis, cirrhosis, and even hepatocellular carcinoma [1]. Although almost all heavy drinkers develop hepatic steatosis, only a small portion progress to advanced liver diseases. Despite many years of extensive research, the cellular and molecular mechanisms underlying the progression of ALD are not fully understood.

Abstinence is likely the best choice for management of ALD in subjects with early disease stages [2]. Classic treatment of ALD includes nutritional support, corticosteroids, and a phosphodiesterase and TNF-*α* (tumor necrosis factor-alpha) inhibitor (pentoxifylline), based on disease severity and other complications [3–5]. Recently, targeting the inflammatory response has received substantial investigative attention. The immunosuppressive drug, prednisolone, and interleukin-22 have been tested in animals and patients with ALD [6, 7]. However, despite intensive studies in the last two decades, there are still no FDA-approved therapies for the treatment of ALD.

The liver acts as the major organ in alcohol metabolism. The oxidative pathway of alcohol metabolism mediated by alcohol dehydrogenase (ADH) and acetaldehyde dehydrogenase (ALDH) generates large amounts of acetaldehyde, which is considered to be the key toxin in alcohol-mediated liver injury [8, 9]. The oxidation of alcohol can also occur via cytochrome P450 2E1 (CYP2E1), which causes tissue injury by the production of reactive oxygen species (ROS) [10, 11].

Although alcohol is mainly metabolized in the liver, it is well known that alcohol consumption causes gut lumen bacterial overgrowth and dysbiosis, intestinal mucosal damage, and increased intestinal permeability, leading to increased translocation of bacteria and their products, endotoxin (mainly lipopolysaccharide, LPS), into the portal circulation. Bacteria and their products stimulate the production of ROS and proinflammatory cytokines and chemokines, resulting in damage to liver cells and the development of liver injury [12, 13]. Gut bacteria-derived endotoxin acts through pattern recognition receptors such as toll-like receptors (TLRs) which are expressed in liver resident macrophages, Kupffer cells, as well as other cell types in the liver. The major endotoxin, LPS, is derived from the cell walls of Gram-negative bacteria in the gut lumen and recognizes TLR4 and its coreceptors, CD14 and MD2, in the liver when penetrating the intestinal barrier and entering into blood stream. Deficiency in the TLR4 complex, such as mutation of TLR4 and lack of CD14 and/or MD2, protects mice from alcohol-induced liver injury. It has been widely demonstrated that alcohol consumption induces endotoxemia [14, 15]. These observations suggest that gut bacteria homeostasis, intestinal barrier integrity, and hepatic TLRs are important in the pathogenesis of ALD.

Therefore, approaches targeting this gut-liver axis may be useful for treating/preventing ALD. In this review, we briefly summarize the recent studies using probiotic intervention for ALD in patients and animal models.

## 2. ALD: Intestinal Dysbiosis and Leaky Gut

Intestinal dysbiosis is defined as an imbalance of the various microbial entities in the intestine with a disruption of symbiosis [16]. Both chronic and acute alcohol consumption lead to bacterial overgrowth and dysbiosis in both the small and large intestine in experimental animals [10, 16–18]. In a rat model of ALD using intragastric gavage feeding, Mutlu and colleagues showed that alcohol ingestion did not change microbiota composition in 4–6-week feeding but significantly altered the mucosa-associated microbiota in the colon after 10-week feeding [14]. A recent study by Yan et al. further demonstrated that 3-week alcohol ingestion in mice led to bacterial overgrowth in the proximal small intestine and dysbiosis, which was associated with the suppression of antimicrobial peptides, Reg3b and Reg3g. The authors also observed an increase in Bacteroidetes and Verrucomicrobia abundance and a decrease in Firmicutes level in alcohol-fed mice. Interestingly, an overgrowth of* Akkermansia muciniphila* was observed in alcohol-fed mice, and this is believed to be responsible for mucin degradation. Moreover, the population of* Lactobacilli* was depleted in alcohol-fed mice [19]. More recently, using metagenomics-based techniques, we observed a decline in the abundance of both Bacteroidetes and Firmicutes phyla, with a proportional increase in the Gram-negative Proteobacteria and Gram-positive Actinobacteria phyla. Genera analysis showed the greatest expansion in Gram-negative alkaline tolerant* Alcaligenes* and Gram-positive* Corynebacterium*. These alterations were accompanied by the changes in colonic pH and liver steatosis [17]. Canesso and colleagues studied the intestinal bacteria composition in germ-free mice and conventional mice after acute alcohol ingestion [18]. This 7-day treatment of alcohol in the drinking water caused a bacterial overgrowth and dysbiosis in conventional mice. Germ-free mice had less fat in the liver after alcohol feeding compared to conventional mice. Moreover, transplantation of intestinal contents from conventional mice to germ-free mice induced inflammation in both intestine and liver.

Intestinal dysbiosis and bacterial overgrowth have also been studied in human alcoholic subjects [20–25]. In 1984, Bode and coworkers found that the bacterial population was increased in the jejunum of alcoholics compared with hospitalized control patients [20]. These same investigators further showed a higher prevalence of small intestine bacterial overgrowth in chronic alcoholics compared to controls using a breath test [21]. These observations were later confirmed by other groups [22, 23]. Recently, Bajaj et al. studied intestinal bacterial composition in 244 alcoholic cirrhotic patients and 25 age-matched controls. Using an index, cirrhosis dysbiosis ratio (CDR, a low number indicating dysbiosis), the authors found that intestinal dysbiosis was more severe in decompensated cirrhotics compared to compensated cirrhotics [24]. Using the lactulose breath test, Gabbard and coworkers observed that moderate drinking was a strong risk factor for small intestine bacterial overgrowth [25].

Recently, Mutlu and colleagues investigated the mucosa-associated colonic microbiome in alcoholics with and without cirrhosis and in controls. Pyrosequencing analysis of colon biopsy samples revealed that mucosa-associated bacteria were persistently altered in a subset of alcoholics, and this was correlated with endotoxemia [26]. This clinical study confirmed the authors' preclinical observation in mice [14].

Taken together, as yet, there is no specific intestinal bacterial pattern identified that has an ethologic role in the development of ALD. However, that fact that alcohol consumption causes bacterial overgrowth and dysbiosis provides an opportunity for the treatment and/or prevention of ALD by targeting intestinal microbiota to prevent dysbiosis and bacterial overgrowth.

## 3. Probiotics and Prebiotics

Probiotics are defined as “live microorganisms which, when administered in adequate amounts, confer a health benefit on the host”, according to the FAO/WHO definition [27]. The beneficial effects of probiotics have been widely investigated in multiple animal models and clinical studies of a variety of disease conditions in the gastrointestinal system such as inflammatory bowel disease, nonalcoholic steatohepatitis (NASH), cirrhosis, and ALD [28, 29]. Ideal probiotic strains for this kind of application should be resistant to bile, hydrochloric acid, and pancreatic juice; be able to tolerate stomach and duodenum conditions and gastric transport; and have the ability to stimulate the immune system, thereby improving intestinal function via adhering to and colonizing the intestinal epithelium. In addition, probiotic strains must be able to survive during manufacture and storage in order to exert considerable healthful outcomes [30]. Currently, the most often used probiotics are* Bifidobacteria,* lactic acid bacteria (LAB),* Propionibacteria*, yeasts (*Saccharomyces boulardii*), and the Gram-negative* Escherichia coli* strain Nissle 1917.* Lactobacilli,* major contributors to the LAB group, are frequently used probiotics. Various species and strains of* Lactobacilli* have been used in the practice in animals and humans, including* Lactobacillus acidophilus*,* Lactobacillus casei*,* Lactobacillus rhamnosus*, and* Lactobacillus helveticus*. Most of these species belong to the phylum Firmicutes*. Bifidobacterium*, which produces lactic acid, is another commonly used probiotic genus and belongs to the Actinobacteria phylum. To date, a large number of probiotics have been reported to be suitable for the treatment of a variety of diseases, and this number is still growing.

Unlike probiotics, prebiotics, which have also been frequently used for disease treatment, are not live bacteria but rather nondigestible carbohydrates. Prebiotics serve as an energy source for “good” bacteria and stimulate the growth and activities of specific bacteria in the gut [19]. The major fermentation products of prebiotics metabolism are short-chain fatty acids (SCFAs), including acetate, propionate, and butyrate. In particular, butyrate has been recognized as a beneficial metabolite associated with many biological functions in the gut. One of the important functions of butyrate is its ability to regulate gene expression through epigenetic mechanisms [31]. Butyrate enhances cell proliferation and inhibits cell apoptosis in normal cells, but not in the transformed cells [32]. A combination of probiotics and prebiotics (synbiotics) is also used in clinical practice and animal models of diseases.

## 4. Probiotics Treatment/Prevention of Experimental ALD

Probiotics are used in experimental animals and, to some extent, in humans, to modulate gut microbial homeostasis and to manage liver diseases including cirrhosis with hepatic encephalopathy, nonalcoholic fatty liver disease (NAFLD), and ALD. A PubMed search using key words “probiotics and alcoholic liver disease” generated 20 publications that described studies using probiotics for management of ALD. Of these, 14 publications were experimental animal studies using several models of ALD (Table 1), including chronic alcohol exposure, single dose acute alcohol exposure, multiple dose alcohol exposure, and alcohol exposure plus LPS challenging. A variety of probiotic strains have also been used, such as* Lactobacillus rhamnosus* GG,* Lactobacillus acidophilus*,* Lactobacillus helveticus*,* Bifidobacterium*,* VSL#3*, heat-killed* Lactobacillus brevis* SBC8803, and* Lactobacillus rhamnosus* GG supernatant.

Among those,* Lactobacillus rhamnosus* GG (LGG) is the most frequently used strain. LGG is a Gram-positive bacterial strain of the* Lactobacillus rhamnosus* species that was isolated in 1983 by Barry R. Goldin and Sherwood L. Gorbach [33]. In several models of ALD in rats and mice, LGG administration showed significant protective effects. LGG reduced plasma endotoxin level, improved liver enzymes alanine transaminase (ALT) and aspartate transaminase (AST), and reduced hepatic steatosis and injury.

Nanji and coworkers were one of the earliest groups demonstrating the effectiveness of LGG in experimental ALD [34]. LGG was administrated to Wistar rats at 10^10^ CFU and reduced alcohol-induced endotoxemia and liver injury. In another study, a combination treatment using* Lactobacillus acidophilus*,* Lactobacillus helveticus*, and* Bifidobacterium* in rats with alcohol pancreatitis-related liver damage effectively protected against endotoxin/bacterial translocation, as well as liver damage in the course of acute pancreatitis and concomitant heavy alcohol consumption [35]. Additional studies using LGG in rats demonstrated reduced alcohol-induced gut leakiness, oxidative stress, and inflammation in both intestine and liver [36] and improved intestinal dysbiosis [14]. Another frequently used probiotic mixture, VSL#3, was shown to be effective in modulating gut microbiota and protecting against alcohol-induced intestinal barrier dysfunction [37].

Recently, our group fed mice with the Lieber-DeCarli liquid diet containing 5% alcohol for 8 weeks to produce hepatic fatty liver and injury. These mice were treated with LGG culture broth at 10^9^ CFU (Colony Forming Unit) for the final 2 weeks along with continued chronic alcohol administration. LGG supplementation reversed established alcoholic hepatic steatosis and injury [38]. This beneficial effect was associated with a reduction in circulating LPS and improved intestinal barrier function mediated, at least in part, by intestinal hypoxia-inducible factor- (HIF-) modulated mucus layer regulation.

## 5. Probiotics Treatment in Patients with ALD

While many reports have studied the effects of probiotics in experimental ALD, clinical trials are limited (Table 2). Stadlbauer and coworkers evaluated the effectiveness of the probiotic* Lactobacillus casei* Shirota on alcoholic cirrhosis (AC) patients (*n* = 12) and healthy controls (*n* = 13) in a small open-labeled study [39]. Compared to control group, cirrhotic patients who received the probiotics for 4 weeks had a significantly lower TLR4 expression and Il-10, sTNFR1 (soluble TNF receptor), and sTNFR2 levels, along with a restored neutrophil phagocytic activity, suggesting that the probiotic is safe and may be effective in the treatment of patients with defective immunity. In a brief report, Loguercio et al. [40] showed that treatment with a synbiotic mixture of different bacteria strains and a prebiotic in 10 AC patients, who were all persistent alcohol users with a median daily intake of pure ethanol of 150 g, significantly improved liver damage and function compared to basal values. Patients were treated with the synbiotic for 2 months, followed by 1 month of a washout period. The ALT and *γ*GT (Gamma Glutamyl Transferase) levels were slightly, but not significantly, increased after the washout period. These results indicate that the effects of synbiotic treatment partially persisted beyond the end of treatment. The same group [41] also reported that a commonly used probiotics mixture, VSL#3, was beneficial in liver disease. This open study involved 22 NAFLD and 20 alcoholic cirrhosis (AC) patients and 36 hepatitis C virus- (HCV-) positive patients with and without liver cirrhosis for comparison. VSL#3 treatment significantly improved plasma levels of malondialdehyde (MDA) and 4-hydroxynonenal (4-HNE) in NAFLD and AC patients, but cytokines (TNF-*α*, IL-6, and IL-10) improved only in AC patients. More recently, Dhiman et al. [42] reported that probiotic VSL#3 treatment reduced liver disease severity and hospitalization in a double-blind trial in patients with cirrhosis including AC (*n* = 89, 46 probiotics, 43 placebos; patients who had alcohol using history in the previous 6 weeks were excluded). Lata and colleagues [43] showed in a double-blind, randomized study that treatment with the probiotic* Escherichia coli* Nissle for 42 days in 34 cirrhosis patients (19 on probiotics; 15 on placebo) who had an alcoholic etiology of their cirrhosis improved colonic colonization and liver function. In an open-labeled, randomized study which involved 66 patients who were diagnosed with alcoholic psychosis and liver disease as well as 24 matched healthy controls, Kirpich et al. [23] demonstrated that, after 5 days of treatment with* Bifidobacterium bifidum* and* Lactobacillus plantarum* 8PA3, mild alcoholic hepatitis patients had a significant end-of-treatment reduction of ALT and AST, lactate dehydrogenase, and total bilirubin. Compared to standard therapy, probiotic treatment significantly reduced serum ALT. This liver function improvement was associated with changes in the fecal commensal bacteria* Bifidobacteria* and* Lactobacilli*.

Taken together, clinical studies suggest that targeting the gut-liver axis through the use of probiotics may have a therapeutic role in the treatment of patients ranging from those with mild alcoholic hepatitis to those with severe alcoholic cirrhosis. As noted, further studies with larger sample sizes for testing the effects of probiotics on ALD are needed. Developing novel probiotic strains and related products, including isolating new probiotic bacteria with improved potency for inhibiting pathogenic bacterial growth, strengthening intestinal barrier function, and improving immunoregulation, and engineered probiotic bacteria producing specific metabolites, will provide more selectivity for treating ALD patients at different disease stages.

Accumulating evidence demonstrates the protective effect of probiotics on multiple pathological disorders. However, these treatments are not always effective because, in many cases, live bacteria must colonize the gut to confer their beneficial effects. The spectrum of pathogenic bacteria varies from patient to patient. Drugs, in particular, antibiotics, used by patients may be harmful to live probiotics. Therefore, an unstable and variable effect of live probiotics may occur. Moreover, the clinically recommended dose of probiotics usually consists of billions of live bacteria. Generally, probiotics are considered safe, but several reports have raised safety concerns about ingesting such large amounts of bacteria, especially when the intestinal function and the patient's immune response are compromised [44–47]. In fact, soluble factors secreted from probiotics and dead probiotics have been used in the treatment of several diseases conditions such as inflammatory bowel disease, colitis, and arthritis [48–50]. Yan et al. demonstrated that soluble proteins produced by probiotic bacteria regulate intestinal epithelial cell survival and growth [51]. Interestingly, the beneficial effects of probiotics on ALD appear to not be restricted to viable probiotic bacteria. Segawa and colleagues demonstrated that oral administration of heat-killed* Lactobacillus brevis* SBC8803 induced the expression of cytoprotective heat shock proteins and improvement of intestinal barrier function leading to amelioration of experimental ALD [52]. Recently, we evaluated the effectiveness of LGG culture supernatant in the prevention of acute and chronic alcohol-induced hepatic steatosis and liver injury [53–55]. Pretreatment with LGG supernatant (LGG-s) reduced hepatic fat accumulation in mice subsequently exposed to acute-binge alcohol [53]. Furthermore, coadministration of LGG supernatant with alcohol in the Lieber-DeCarli liquid diet for 4 weeks significantly prevented alcohol-induced intestinal barrier dysfunction, endotoxemia, fatty liver, and inflammation in mice [54, 55]. The use of probiotic culture supernatant opens a new avenue for the probiotic application. Further characterization of the LGG-s active components will enhance our understanding of the protective effect of probiotics in ALD and advance the development of new therapeutic strategies for ALD.

## 6. Potential Mechanisms of Probiotics in ALD

**Figure 1:**
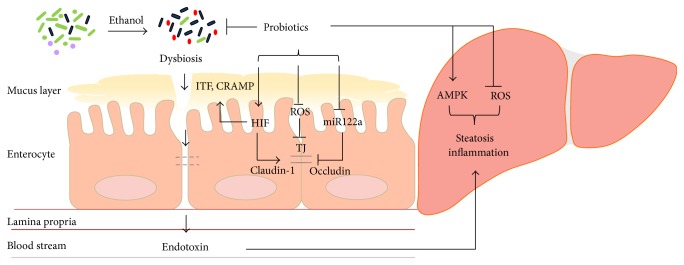
Proposed mechanisms of probiotic function in ALD. Ethanol consumption causes a gut bacterial overgrowth and a dysbiosis leading to impaired mucus layer and dysfunctional tight junctions. The damaged epithelial barrier function results in endotoxemia. Elevated endotoxin activates Kupffer cells in the liver and induces hepatic steatosis and inflammation. Probiotics and related products prevent ethanol-induced effects in the intestine and the liver through multiple mechanisms: (1) positive modification of gut microbiota; (2) reduction of ROS production in intestine and liver; (3) enhancement of mucus layer component, ITF, and antimicrobial peptide, CRAMP, and tight junction protein claudin-1 expression through increased HIF signaling; (4) inhibition of miR122a expression leading to occludin upregulation; and (5) activation of hepatic AMPK.

Despite many proof-of-effectiveness studies of probiotics on the treatment of both experimental and human ALD, the mechanisms by which probiotics function are still poorly understood. To date, several important mechanisms including the modification of gut microbiota, improvement of the intestinal epithelial barrier function, regulation of the immune system and inflammation, and alteration of hepatic lipid homeostasis have been proposed. These mechanisms involve gene expression regulation in both intestinal and hepatic tissues.  Figure 1 summarizes many of the proposed mechanisms of probiotic function in ALD.

**Figure 2:**
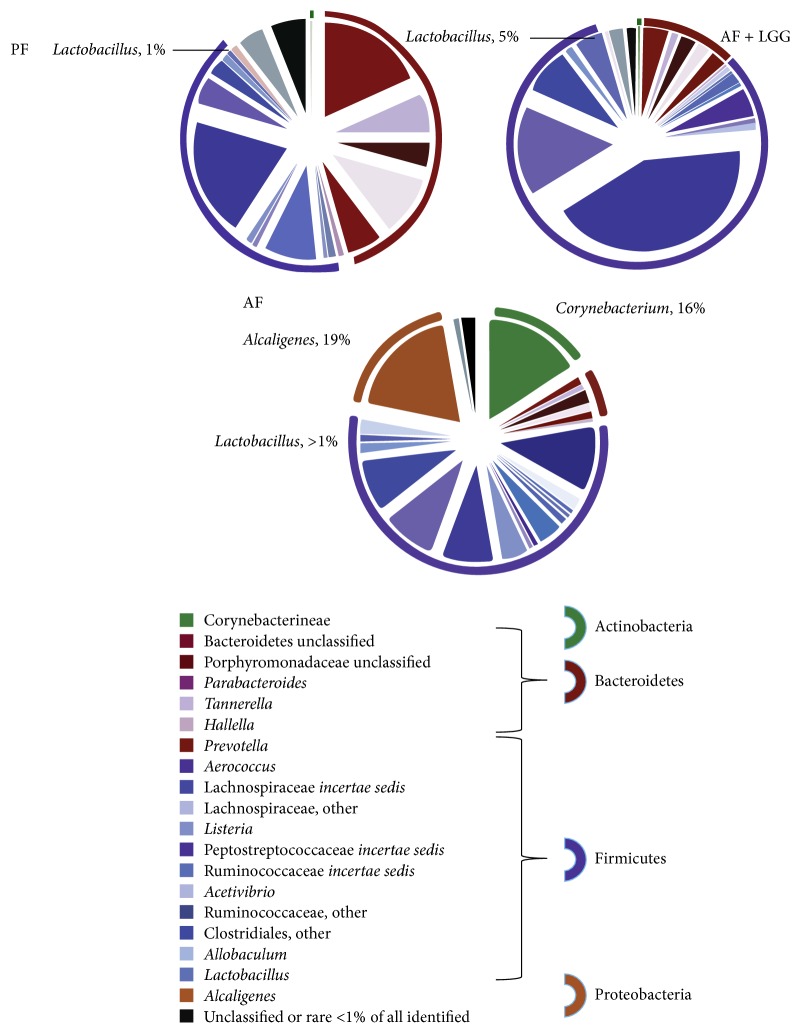
The relative distribution of the bacterial phyla and genera in response to ethanol feeding and LGG supplementation. Mice were fed with Lieber-DeCarli diet containing 5% EtOH or pair-fed with maltose dextrin for 6 weeks.* Lactobacillus rhamnosus* GG was supplemented at a dose of 10^9^ CFU/day for the last 2 weeks with continued alcohol feeding. The fecal samples were analyzed by a metagenomic approach. The microbiome of the PF, AF + LGG, and AF mice is shown in the pie charts and color coordinated by genus and phylum. The different shades of color represent the different genera and the common color spectrum (reds, purples, green, and orange) represents the phyla. The outer ring around the pie charts also depicts the different phyla. The microbiome of AF mice is characterized by greater abundance of* Alcaligenes* and* Corynebacterium* and loss of* Tannerella*. The AF + LGG group shows a much greater abundance of* Lactobacillus* and nonspecific Ruminococcaceae* incertae sedis* compared to the other exposure groups (PF: pair-feeding; AF: alcohol feeding; and AF + LGG: alcohol feeding plus* Lactobacillus rhamnosus* GG, adapted from [17]).

Alterations of gut microbiota have been recognized widely as one of the major mechanisms underlying probiotic function. One of the first studies in rats with ALD showed a dysbiosis in colon lumen contents, which was prevented by probiotic and prebiotic treatment [14]. Several other studies also demonstrated that supplementation with probiotics restored gut microbiota homeostasis and alleviated alcohol-induced liver injury [17, 19, 23, 40, 42, 43]. We have shown that, in mice fed with a 6-week course of alcohol plus 2-week treatment with LGG with continued alcohol intake, the LGG positively modified the alcohol-induced dysbiosis [17]. Chronic ethanol feeding caused a decline in the abundance of both Bacteroidetes and Firmicutes phyla, with a proportional increase in Proteobacteria and Actinobacteria phyla. Gram-negative alkaline tolerant* Alcaligenes* and Gram-positive* Corynebacterium* were the bacterial genera that showed the greatest expansion. In parallel with the qualitative and quantitative alterations in the microbiome, ethanol caused an increase in plasma endotoxin, fecal pH, hepatic inflammation, and injury. Notably, the ethanol-induced pathogenic changes in the microbiome and the liver were prevented by LGG supplementation [17] (Figure 2). Clearly, due to the critical role of microbiota in gut-liver axis, restoration of gut microbiota contributes to the beneficial effects of probiotics in ALD.

One of the major functions of gut bacteria is to metabolize food to produce metabolites that are beneficial (or harmful in the case of harmful bacteria) to the host. In our recent study [56] using a metabolomics approach, we demonstrated that heptadecanoic acid (C17:0), a long chain fatty acid produced only by bacteria, was reduced by alcohol ingestion and increased by probiotic treatment. Interestingly, supplementation of heptadecanoic acid attenuated ALD in mice [57]. Moreover, short-chain fatty acids, which have multiple roles in the intestine including serving as energy source and immunoregulation, were reduced by alcohol and increased by probiotics [58–60]. We also showed that probiotic supplementation normalized the abundance of several amino acids in the liver and in the gut [56]. These results demonstrate that LGG-s attenuates ALD by mechanisms involving increasing intestinal fatty acids and amino acid metabolism.

Gut barrier function and endotoxemia are at the center of gut-liver axis in multiple disease conditions. Probiotic administration has been shown to reinforce the intestinal barrier and reduce endotoxin levels in both NAFLD and ALD. The intestinal epithelial barrier is a complex system composed of cellular, physical, and chemical components [61]. The epithelial cells form a lining with the paracellular space sealed by tight junctions (TJ) and adherens junctions [62], and this is covered by a protective mucin layer that physically blocks most particles from direct contact with the epithelial cells [63]. Alcohol consumption, both acute-binge and chronic, directly affects the gut intestinal barrier at multiple levels including tight junctions, production of mucin, and recruitment and activation of inflammatory cells to the intestinal wall [64]. Our studies evaluated the effects of probiotics LGG and LGG-s on epithelial cell permeability and severity of hepatic steatosis using* in vivo* (mouse) and* in vitro* (epithelial cell culture) models [38, 53, 55]. Probiotics administration increased the expression of tight junction proteins claudin-1, ZO-1, and occludin at both protein and mRNA levels and normalized barrier function by decreasing intestinal permeability using* ex vivo* measurement in the ileum or transepithelial electrical resistance (TEER) in Caco-2 monolayers. In addition, we also showed that LGG and LGG-s restored the expression of mucus-related genes including intestinal trefoil factor (ITF), as well as P-glycoprotein (P-gp), and cathelin-related antimicrobial peptide (CRAMP), which were decreased by alcohol ingestion in mice.

How do probiotic bacteria affect gut barrier function? Gut bacteria metabolize ethanol to acetaldehyde by cytochrome P450 2E1 (CYP2E1) that produces a large number of reactive oxygen species (ROS), which could damage intestinal barrier components including mucus layer and tight junctions. A recent study also demonstrated that bacterial metabolism produces endogenous ethanol, which might also have deleterious effects on the gut barrier [65]. Probiotics, therefore, could contribute to intestinal barrier function by modulating certain gut bacteria leading to reduced metabolism of alcohol and ROS production in the intestine. Intestinal inflammatory cells such as mast cells also affect alcohol-induced epithelial barrier dysfunction [66]. Alcohol-induced barrier dysfunction is associated with local and systemic production of proinflammatory cytokines such as TNF-*α* and IL-1*β*. Several studies showed that probiotic administration decreased alcohol-induced systemic and intestinal TNF-*α* and IL-1*β* levels [37, 52, 67], which might contribute to the beneficial effects of probiotics on gut barrier integrity in ALD.

The intestinal mucosa experiences profound fluctuations in blood flow and metabolism. Alcohol metabolism in the intestine could cause tissue hypoxia that triggers induction of a master transcription factor, hypoxia-inducible factor (HIF). HIF is important for maintaining barrier function by increasing global mucosal protective mechanisms including mucin production and stabilization via regulation of ITF, xenobiotic clearance by P-gp, and various other nucleotide signaling pathways [68]. However, alcohol-induced ROS could damage this compensatory role of HIF leading to barrier dysfunction [38]. LGG administration restored intestinal HIF expression and function in ALD in mice. In addition, the intestinal level of another important HIF target, CRAMP, was decreased by alcohol exposure and increased by LGG-s treatment in mice, implying a potential role of probiotics in the regulation of gut microbiota in ALD [53]. Additional studies reported that antimicrobial proteins Reg3g and Reg3b were downregulated by chronic alcohol exposure, which may contribute to the quantitative and qualitative changes in the gut flora, and prebiotics treatment can partially restore Reg3g levels, leading to decreased intestinal bacterial overgrowth, and ameliorates alcoholic steatohepatitis [19]. A recent study identified one of the major tight junction molecules, claudin-1, as being a HIF transcriptional target suggesting that probiotics may protect the gut barrier directly through the HIF-tight junction axis [69].

Tight junction proteins are regulated by multiple mechanisms. Ye et al. demonstrated that intestinal occludin is a target of microRNA 122a [70]. TNF-*α* induced an increase in miR122a leading to a reduction of intestinal occludin protein expression. Similarly, alcohol ingestion increased miR122a levels in the intestine. Probiotic LGG-s administration decreased miR122a levels and therefore increased occludin expression [54].

In addition to intestinal mechanisms in ALD, probiotic bacteria also act on the immune system through TLRs. We have shown that two weeks of LGG supplementation reduced hepatic inflammation and markedly reduced TNF-*α* expression in a murine model of ALD. We also demonstrated that, in an* in vitro* system using human peripheral blood monocytes-derived macrophages, incubation with ethanol primes, both lipopolysaccharide- and flagellin-induced TNF-*α* production, and LGG-s reduced this induction in a dose dependent manner [71].

In a recent study [55], we further demonstrated that probiotics may function as a direct mediator in regulating hepatic lipid metabolism and apoptotic cell death. LGG-s administration prevented alcohol-increased expression of genes involved in lipogenesis and alcohol-decreased genes involved in fatty acid *β*-oxidation. Importantly, these lipid regulatory effects were mediated through probiotic action on adenosine-monophosphate-activated protein kinase (AMPK) phosphorylation. LGG-s also decreased Bax expression and increased Bcl-2 expression, which attenuated alcohol-induced hepatic apoptosis. Thus, probiotics likely exert their beneficial effects, at least in part, through modulation of hepatic AMPK activation and Bax/Bcl-2-mediated apoptosis in the liver.

Myosin light-chain kinase (MLCK) is a downstream target of TNF-*α*. MLCK can be phosphorylated in intestinal epithelial cells after alcohol consumption, thus playing a vital role in regulation of the epithelial barrier integrity. Ma et al. [72] found that ethanol can stimulate MLCK activation and monolayer permeability in Caco-2 cells, which can be effectively inhibited by the MLCK inhibitor, ML-7. A similar finding was demonstrated by Su and coworkers [73] using MLCK intestinal epithelial specifically transgenic (Tg) mice in a colitis model. Tg mice demonstrated significant barrier loss and a more severe form of colitis than controls. Recently, Chen et al. [74] further demonstrated the partial contribution of MLCK to intestinal barrier dysfunction and liver disease after chronic alcohol feeding using MLCK-deficient mice. Whether probiotics exert their beneficial effects through inhibition of MLCK in ALD has not been demonstrated yet, but a newly published study by Sun and coworkers [75] indicated that* Lactobacillus acidophilus* treatment of traumatic brain injury (TBI) mice can efficiently prevent the damage of interstitial cells and improve the terminal ileum villus morphology via decreased MLCK concentration.

## 7. Conclusion

In conclusion, with the growing body of studies demonstrating that ALD is closely associated with gut microbial alterations and that gut bacteria/bacterial products play an important role in ALD progression, using probiotics for the prevention and/or treatment of ALD continues to attract more investigative and clinical attention. Although increasing numbers of probiotic strains and related products have been identified as being useful in ALD, the precise mechanisms underlying the role of probiotics in regulating gut microbiota, intestinal barrier function, and eventfully alcoholic liver disease need further investigation. It is likely that probiotics work through multiple mechanisms. Specific actions may be particularly important in specific disease processes and individual people; thus, this may be a unique form of personalized medicine.

## Figures and Tables

**Table 1:** Probiotics application in experimental ALD.

Animal	Alcohol feeding model	Probiotics/(prebiotics) treatment	Effect	Mechanism based on the study	Reference
Rat	Liquid diet containing ethanol and corn oil for 1 month	A daily bolus of *Lactobacillus rhamnosus* GG at 10^10^ CFU/mL for 1 month	Improved liver pathology score and lowered plasma endotoxin level	Prevention of endotoxemia, improved barrier and immune function	[34]

Rat	15 g/kg/day ethanol consumption for 2 weeks	Liquid diet through an intragastric tube containing *Lactobacillus acidophilus*, *Lactobacillus helveticus*, and *Bifidobacterium* pretreatment for 1 week	Normalized AST/ALT levels, improved liver histological score, and lowered plasma endotoxin level	Prevention of endotoxemia, improved barrier and immune function	[35]

Mouse	Lieber-DeCarli diet (5% EtOH, w/v) for 4/5 weeks	Heat-killed *Lactobacillus brevis* SBC8803 orally administered at 100/500 mg/5 mL/kg/day for 5 weeks	Reduced serum ALT and AST, TG, and liver total cholesterol	Reduction of gut-derived endotoxin through induction of heat shock protein	[52]

Rat	Gavage with gradually increased ethanol concentration to 8 g/kg/day in 10 weeks	Gavage with *Lactobacillus rhamnosus* GG at 2.5 × 10^7^ CFU/mL/day or oats 10 g/kg/day for 10 weeks	Normalized colonic microbiota composition, reduced hepatic steatosis, and improved alcoholic steatohepatitis	Prevention of colonic mucosa-associated dysbiosis, reduction of oxidative stress in intestine and liver	[14, 36]

Mouse	Lieber-DeCarli liquid diet (5% EtOH, w/v) for 8 weeks	Culture broth of *Lactobacillus rhamnosus* GG added to the diet at 10^9^ CFU/mouse/day for the last 2 weeks	Reduced plasma ALT, endotoxin level, liver steatosis, and inflammation	Increasing HIF-mediated mucosal protecting factors and tight junction proteins, positive modification of gut microflora, reduction of endotoxemia, and desensitization of macrophage to endotoxin	[17, 38, 71]

Mouse	Acute binge, one dose of 6 g/kg ethanol	Gavage with *Lactobacillus rhamnosus* GG supernatant at a dose of equivalent to 10^9^ CFU/mouse/day pretreatment for 5 days	Reduced liver enzymes, hepatic steatosis, hepatic ROS, and serum endotoxin level	Increasing HIF-mediated mucosal protecting factors and intestinal tight junction proteins	[53]

Rat	3 doses of 5 g/kg ethanol administration every 12 hours	Intragastric feeding of VSL#3/heat-killed VSL#3 at 0.6 g/kg body wt. pretreatment for 30 min	Lowered plasma endotoxin level	Regulation of the ecological balance of gut microbiota, prevention of TNF-*α*, decreasing epithelial permeability, and increasing tight junction proteins—ZO-1, occludin	[37]

Mouse	Multiple doses of 5 g/kg/day ethanol, plus multiple LPS for a total of 11 weeks	Intragastric feeding of *Lactobacillus rhamnosus* R0011 and *Lactobacillus acidophilus* R0052 (1 mg/mL/day) for the last 2 weeks	Improved hepatitis activity, increased body weight	Modulation of the gut-liver axis: reduction of ALT, TLR4, TNF-*α*, and IL-1*β* expression; increasing IL-10 expression	[67]

Mouse	Lieber-DeCarli liquid diet (5% EtOH, w/v) for 4 weeks	Liquid diet containing *Lactobacillus rhamnosus* GG supernatant at a dose of equivalent to 10^9^ CFU/mouse/day pretreatment for 4 weeks	Reduced hepatic steatosis and inflammation, reduced endotoxemia; normalized fatty acid levels in mouse liver and feces	Restoration of occludin in ileum mediated by the inhibition of miR122a expression, increasing hepatic AMPK activation, and inhibition of hepatic apoptosis; increasing intestinal and decreasing hepatic fatty acid and increasing amino acid concentration	[54–56]

**Table 2:** Probiotics in ALD—clinical evidence.

Disease	Treatment and duration	Observations	Reference
Alcoholic cirrhosis patients, *n* = 10	VSL#3 treatment for 3 months	Reduced plasma ALT, AST, and GGT levels; normalized plasma TNF-*α*, IL-6, and IL-10 levels; and decreased MDA, 4-HNE, and S-NO levels	[40]

Alcoholic cirrhosis patients, *n* = 20	*Lactobacillus casei* Shirota for 4 weeks of treatment	Normalized phagocytic capacity, decreased TLR4, sTNFR1, sTNFR2, and IL10 levels	[41]

Alcoholic cirrhosis patients, *n* = 34	*Escherichia coli* Nissle for 42 days of treatment	Improvement in intestinal colonization, restored microflora in feces, and reduced endotoxin levels in blood	[43]

Alcoholic cirrhosis patients, *n* = 12	A mixture of different lactic acid bacteria strains treated for 2 months	Positive effects on ecological balance of enteric commensals, reduced ALT, *γ*-GT, and TNF-*α* levels	[39]

Patients with alcoholic psychosis and liver disease, *n* = 66	*Bifidobacterium bifidum* and *Lactobacillus plantarum *8PA3 for 5 days of treatment	Increased numbers of both *Bifidobacteria* and *Lactobacilli*; reduction in ALT, AST, GGT, LDH, and total bilirubin	[23]

Alcoholic and nonalcoholic cirrhosis and hepatic encephalopathy patients *n* = 89	VSL#3 treatment for 6 months	Reduced risk of hospitalization for HE (hepatic encephalopathy), improved CTP (Child-Turcotte-Pugh) and MELD (model for end-stage liver disease) scores	[42]
